# Supplementary material for: Assessing Tumour Haemodynamic Heterogeneity and Response to Choline Kinase Inhibition Using Clustered Dynamic Contrast Enhanced MRI Parameters in Rodent Models of Glioblastoma
Source: Cancers (Basel). 2022 Feb 26;14(5):1223. doi: 10.3390/cancers14051223 (PMC8909848; doi:10.3390/cancers14051223)
Supplement: Supplementary file 1 [file cancers-14-01223-s001.zip › cancers-1542934-supplementary.pdf]

Supplementary Material: Assessing tumour haemodynamic heterogeneity and response to choline kinase inhibition using clustered dynamic contrast enhanced MRI parameters in rodent models of glioblastoma

A1. *DCE data analysis*

Standard perfusion parameters, such as K^{trans} and v_e can be estimated by fitting the contrast agent concentration $C_t(t)$ to the TM [24] equation 1,

$$C_t(t) = K^{trans} \int_0^t C_p(\tau) e^{-K^{trans}(t-\tau)/v_e} \, d\tau \quad (1)$$

Where, $C_p(\tau)$ is the contrast agent concentration in the blood plasma, which is the AIF. The ratio between K^{trans} and v_e results in the third pharmacokinetic parameter K_{ep} (unit: 1/min). The ETM [24] expands Eq. 1 by including the plasma volume fraction (v_p) and is described as

$$C_t(t) = K^{trans} \int_0^t C_p(\tau) e^{-K^{trans}(t-\tau)/v_e} \, d\tau + v_p C_p(t) \quad (2)$$

The model described in Eq. 2 assumes that equilibrium transcytolemmal water exchange between the intracellular space and extracellular extravascular space is infinitely fast. Water exchange between the intracellular space and the extracellular space affects the degree of T1 shortening due to the administration of contrast agent. To account for this effect, the SSM was used to estimate the mean intracellular water molecule lifetime, τ_i (unit: sec) [13, 25] using the equation below (Eq. 3):

$$R_L(t) = \left(\frac{1}{2}\right)\left\{2R_{1i} + r_{1o}[C_t(t)] + \frac{\left(R_{10} - R_i + \frac{1}{\tau_i}\right)}{p_o}\right\} - \left(\frac{1}{2}\right)\left\{\left(\frac{2}{\tau_i} - r_{10}[C_t(t)] - \frac{\left(R_{10} - R_i + \frac{1}{\tau_i}\right)}{p_o}\right)^2 + \frac{4(1-p_o)}{\tau_i^2 p_o}\right\}^{\frac{1}{2}} \quad (3)$$

Where, $R_L(t)$ is the relaxation rate constant of the SSM. R_i is the intracellular rate constant in the absence of exchange of contrast agent. R_{10} is the pre-contrast relaxation rate, and r_{1o} is the relaxivity of the contrast agent. The SSM2 model accounts for the contribution of the contrast agent from the plasma compartment. This includes the intravascular water molecule lifetime. This model is combined with the ETM for estimation of the pharmacokinetic parameters utilising an exchange matrix ($\boldsymbol{X}$) [29]:

$$\boldsymbol{X} = \begin{pmatrix} -(R_b + k_{bo}) & k_{ob} & 0 \\ k_{bo} & -(R_{10} + k_{ob} + k_{oi}) & k_{io} \\ 0 & k_{oi} & -(R_i + k_{io}) \end{pmatrix}$$

Where R_b is the intravascular rate constant in the absence of exchange of contrast agent, k_{bo} (= $1/\tau_b$) represents the transfer of water from blood to interstitium; k_{io} (= $1/\tau_i$) the transfer of water from the intracellular space to the interstitium. k_{ob} represents the transfer of water between the interstitium to blood and k_{oi} the transfer between interstitium to intracellular space.

The 2CXM model (Eq. 4) considers the plasma and interstitium as single compartments and provides the additional parameters: permeability-surface area product (PS) and plasma flow (F_p), both measured in mL/min/100 mL and is described in [26, 27]:

$$C_t(t) = F_+ \int_0^t C_p(\tau) e^{-K_+(t-\tau)}\, d\tau + F_- \int_0^t C_p(\tau) e^{-K_-(t-\tau)}\, d\tau \qquad (4)$$

where, $K_\pm = \frac{F_\mathrm{p}}{(v_\mathrm{p}+v_\mathrm{e})\tau_\mp}$, $F_\pm = \pm F_\mathrm{p} \frac{\tau_\pm - 1}{\tau_+ - \tau_-}$, $\tau_\pm = \frac{E - Ee + e}{2E}\left\{1 \pm \sqrt{1 - 4\frac{Ee(1-E)(1-e)}{(E-Ee+e)^2}}\right\}$, $E = \frac{PS}{PS+F_\mathrm{p}}$ and $e = \frac{v_\mathrm{p}}{v_\mathrm{p}+v_\mathrm{e}}$. The K^{trans} is finally calculated in Eq. 5 as:

$$K^{trans} = \frac{F_p . PS}{F_p + PS} \qquad (5)$$

Table S1. Pearson's correlation (*r*) for tumour volume vs mean of parameters.

	TM	ETM	SSM	2CXM	SSM2
$\boldsymbol{K^{trans}}$	0.17	0.14	0.2	0.13	0.11
$\mathbf{K_{ep}}$	0.03	–0.13	0.01	–0.1	–0.01
$\boldsymbol{v_e}$	0.28	0.33	0.29	0.32	0.26
$\boldsymbol{v_p}$	N.A	0.28	N.A	0.18	0.11
$\boldsymbol{\tau_\mathrm{i}}$	N.A	N.A	0.15	N.A	0.1
$\boldsymbol{F_p}$	N.A	N.A	N.A	0.11	N.A

Note. TM, Tofts model; ETM, extended Tofts model; SSM, Shutter speed model; 2CXM, two-compartment exchange model; SSM2, second generation Shutter speed model; N.A, not applicable.

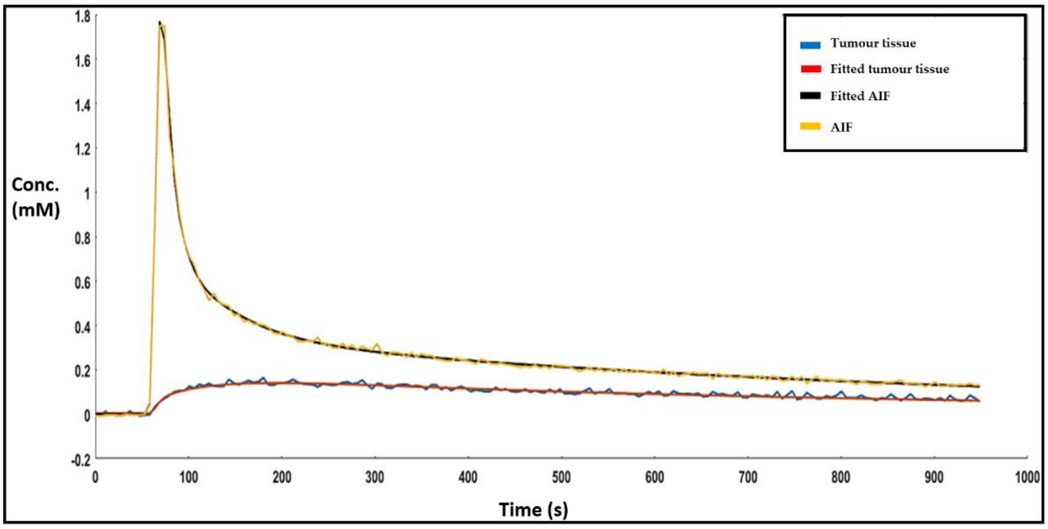

Figure S1. An example of fitting tumour tissue (mean across tumour ROI) from F98 rat GBM (Original: blue, fitted: red) with SSM and the AIF (yellow) is fitted using hybrid bi-exponential and gamma variate model fitting.

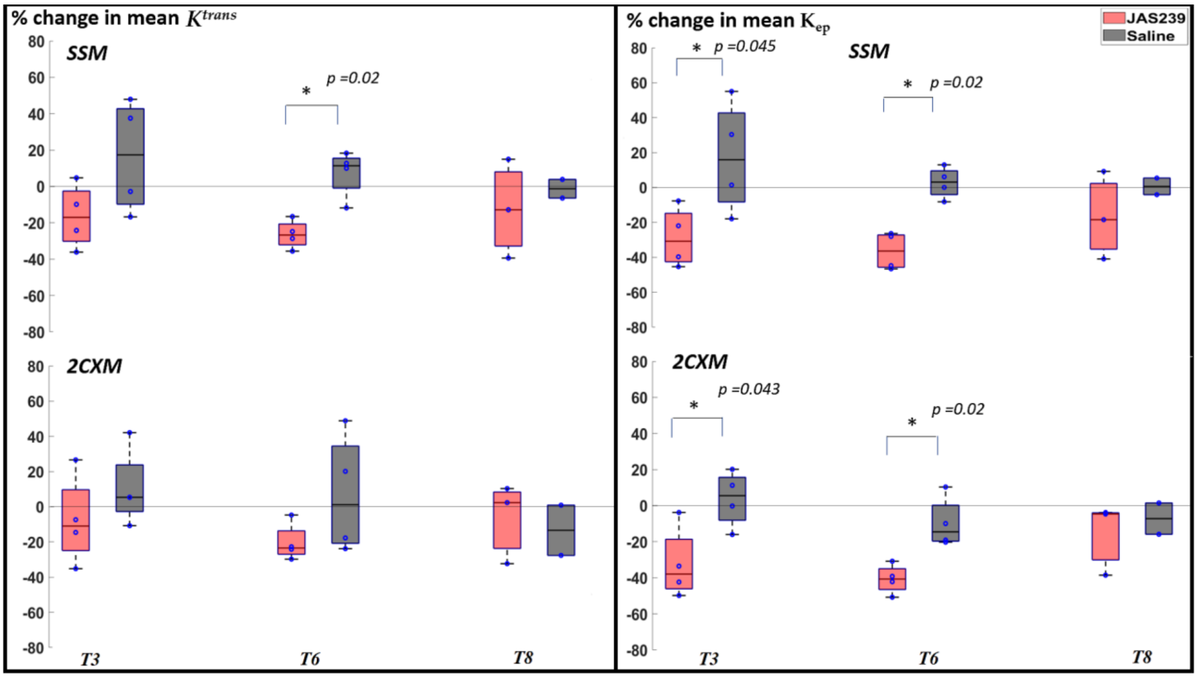

Figure S2. Box plot for percentage change (with respect to baseline) in mean K^{trans}(left: top and bottom) in F98 rat GBM with JAS239 (red) and saline (grey) treatment at different time points using the SSM and 2CXM models, respectively. The differences in the mean K_{ep} are shown in the right (top and bottom).

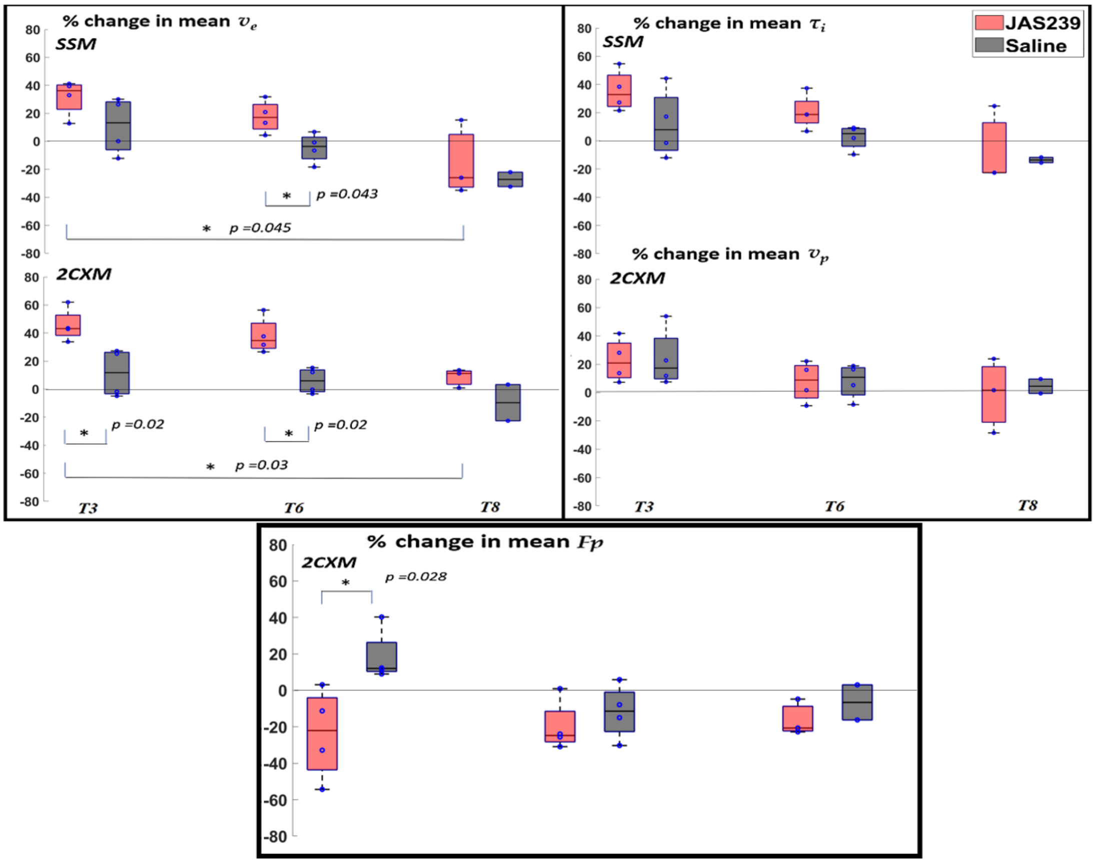

Figure S3. Box plot for percentage change (with respect to baseline) in mean v_e (left: top and bottom) in F98 rat GBM with JAS239 (red) and saline (grey) treatment at different time points using the SSM and 2CXM models, respectively. The same for mean τ_i (right: top) using SSM. Box plot for percentage change in mean v_p (right: bottom) with JAS239 (red) and saline (grey) treatment at different time points using the 2CXM model. The same for mean F_p (bottom) using the 2CXM.

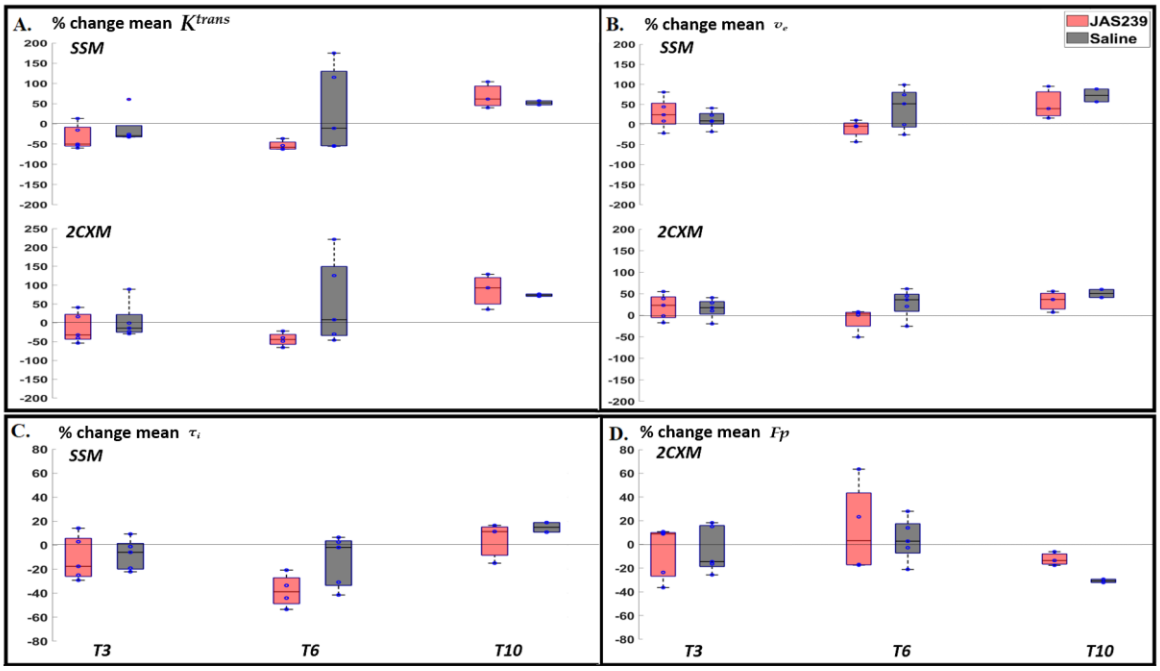

Figure S4. Box plots showing changes (with respect to baseline) in DCE parameters in GL261 mice GBM with JAS239 treatment **A.** Percent change in mean K^{trans} with respect to baseline values in JAS239 (red) and saline (grey) treatment at different time points using the SSM and 2CXM models. **B.** Same for v_e **C.** mean τ_i using SSM, or **D.** mean F_p using 2CXM.

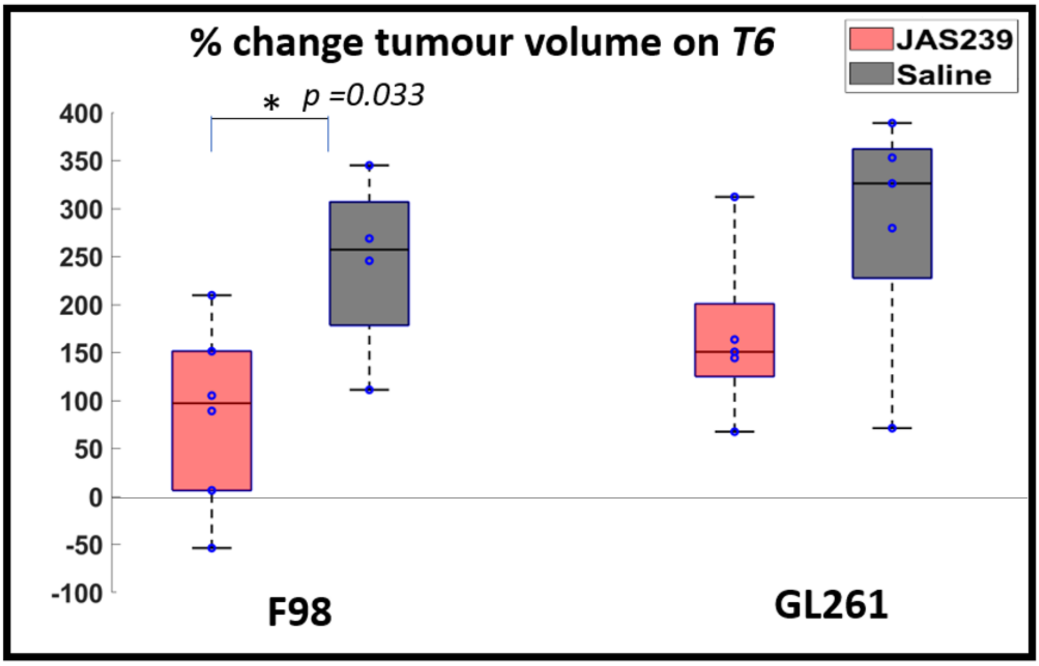

Figure S5. Box plots comparing percentage change (with respect to baseline) in tumour volume between JAS239 (red) and control (grey) groups in F98 rat and GL261 mice GBMs.
